# The Hospital Nursing Supplement

**Published:** 1894-08-25

**Authors:** 


"The Hospital" Nursing Mirror.

Being the Extra Nursing Supplement of "The Hospital" Newspaper.

[Contributions for this Supplement should be addressed to the Editor, The Hospital, 428, Strand, London, W.C., and should have the word "Nursing" plainly written in left-hand top corner of the envelope.]

### News from the Nursing World.

### OUR CHRISTMAS COMPETITIONS.

We want very much to have a large number of competitors this year, and to be able to send, by their kind co-operation, a handsome supply of warm garments to patients in the hospitals next Christmas Day. Our parcels have always been warmly welcomed and highly appreciated, and our readers would feel fully repaid for their exertions if they realised the comfort which warm garments ensure to delicate and ill-clad convalescents exchanging the comfortable ward for chilly streets and poor homes at the most trying season of the year. The prizes offered are 20s. for the most serviceable dressing gown; 10s. for the best flannel shirt; 7s. 6d. for the best flannel petticoat; 7s. 6d. for the best over petticoat; 7s. 6d. for the best bed jacket; 5s. for the best knitted pair of men's socks; and 2s. 6d. for the second best pair.

### PRUDENT PRECAUTIONS.

Amongst the hospitals which are placing wards in readiness for cholera patients the Seamen's Hospital, Greenwich, the Gravesend Cottage Hospital, and Guy's Hospital, have been mentioned. The Metropolitan Asylums Board can receive 1,700 patients in their floating infirmaries and shelters—therefore no epidemic will find England unprepared. We trust that cholera will be kept effectually at bay by the excellent precautionary measures of the health authorities. Amongst these must be mentioned the practical advice issued to householders on sanitary matters and the gratis provision of disinfectants. The order that all cases of diarrhœa are to be reported to the sanitary officer should be borne in mind by district nurses who have so much influence with the people they work amongst. They have also large opportunities of seeing what needs special attention in the homes of the poor. A friendly word of warning from the nurse, whose value they have learnt, is seldom taken amiss, and it may even sometimes prove more acceptable than any printed rules.

### A COTTAGE HOSPITAL APPRECIATED.

The report of the North Argyll Nursing Association and Cottage Hospital shows that in the year ending August 1st, ninety persons were nursed in the hospital, as against fifteen in the previous year. This, as the Secretary remarks, is a clear proof of the esteem in which good nursing is held in the district; and an appeal for increased financial support and for gifts of linen, clothing, books, &c., can hardly fail to receive a hearty response.

### WORTHY OF IMITATION.

Those feeling depressed by the recent revelations regarding neglected paupers would do well to go to Greenwich and ask leave to see over that workhouse infirmary. They will find how much is done already in some places for the sick and helpless. Bright, clean wards, with many windows and good beds, small red rugs across the foot of each; flowers on the tables, the mug of tea and plate of bread and butter, neatly served on a tiny wooden tray to each bed-ridden patient. Those who can sit up take their meal at a long table covered by a snowy cloth, all betokening a model hospital quite free from that terrible air of bald pauperism which, alas! is still prevalent elsewhere. Cheerful ward kitchens are refined into pleasant semblance of the "nurses' parlour," and tiny safes for the rations to be kept outside in, look as wholesome as they are homelike. The children's ward is clean and pretty, with a *Truth* doll conspicuous in the centre, and the best ornaments of all, a group of well-cared-for little patients taking their evening meal together on a rug. A small table in front of the fireplace is the favoured spot in winter for the convalescents, and the grate is so safely railed round that no mishap can accrue from close proximity to the attractive blaze. The neat tiled room adjoining the admission room makes the ordeal, to a patient, of the first bath, seem but a pleasant experience, the admission room itself being also bright and cosy. Certainly any man or woman doubting the advantage of trained nursing, combined with a fine modern building, would soon be convinced of its success in the beautiful wards at Greenwich, where courtesy and kindliness seem to season the treatment of the sick, the desolate, and poor.

### DEGRADING THE CHILDREN.

"There's a little boy and girl at the door, ma'am," announces the maid, "and they want you to give them a trifle; they say it's for the poor little things to get a day in the country." This is the form taken by one of the many begging nuisances of the day. It's just about as "genuine," too, as the "fireman," who comes round with his book for subscriptions, or the precocious little boy who boards the omnibus with his amateur collecting-box, often bearing an illiterate label which ought to deceive no one into believing it the offspring of any hospital whatever. Even if the book or the box be connected with any lawful agency the fact of confiding it to a child is reprehensible. In plain English it means an encouragement to the little ones to beg, and it fosters their least commendable instincts. It is unfair to take advantage of the fact that a child's petition is almost irresistible when addressed to an average adult. This result can in no way justify the means employed, to our thinking. The evil would soon be abolished if donations were systematically sent to the headquarters of charities, and indiscriminate collecting cards and street boxes entirely discountenanced.

### RECOMMENDATIONS "EMPHASIZED."

The inquests held at Oldham on two of the patients have brought to light some serious defects in the nursing at the workhouse infirmary. There is no superintendent of nursing, the staff is inadequate, and at night the three blocks of buildings, containing two hundred patients, are left in charge of a single nurse. The Guardians cannot plead ignorance of the defects in their arrangements, for the local press shows that in 1892 Dr. Downes, of the Local Government Board, drew attention to the requirements which the coroner, as well as the medical officer, now urge upon their notice. Dr. Downes recommended that a charge nurse and two probationers should be told off for night duty, and that a superintendent should be appointed and additional probationers. The nursing staff was increased after this, but not adequately. It is to be hoped that due consideration will be attached to the opinion of the jurors at the recent inquest, "that the Board of Guardians be notified that, in their opinion, the nursing at the workhouse infirmary is insufficient, especially in the night, and the employment of inmates as nurses in such cases as this is unsatisfactory." It would be unjust to say that the cases of suicide and of death from poison were altogether due to the insufficient number of nurses, but it is a disastrous coincidence that they should have occurred during the hours when one unfortunate woman was put in sole charge of two hundred sick and infirm paupers. The propositions of the medical officers seem likely to receive due consideration from some of the Guardians, and the others, who appear at present somewhat ruffled by recent criticism will no doubt see the wisdom of making arrangements which will exonerate them from blame in future.

### TIME OFF DUTY.

There is an article in the *Westminster Review* on "The Nursing in Poor Law Infirmaries," which brings forcibly to public notice another matter needing reform in some, at any rate, of these institutions. We are told that "the daily routine of a nurse's life in an infirmary is much the same as it is elsewhere, except that in some institutions the off-duty time is very liberal in comparison." As an illustration of this, we read that at Chelsea Infirmary "each nurse is allowed four afternoons from two to five one week, and the next week a half-day from two to ten, and, in addition to this, a part of every Sunday." This appears to imply that during the week in which the "half-day" is given the nurse has to work the whole of five days, "and pretty hard, too," and in the alternate week two whole days pass without any time being allowed off duty. If this off-duty time is "very liberal" in comparison with any infirmaries in the present day, it seems inevitable that the Local Government Board should soon add another clause to their valuable "recommendations," and deal with this matter. If any hospitals with training schools are amongst the "institutions" referred to by the writer of this interesting article, it would be well that their names should be made public, in order that intending probationers should learn in time where not to go!

### A GOOD WOMAN.

"She was a truly good woman, who did a great deal of real work unobtrusively," says one who knew Miss Jane Thompson well. "A kinder heart never beat," "A true friend to nurses," are other comments on her who has gone to rest. Miss Thompson was one of the best known and most respected women in Dublin, and a large number of her friends were present on the occasion of the meeting presided over by the Archbishop of Dublin in the Palace, for the object of considering the most suitable form for the proposed memorial. "The indefatigable zeal with which Miss Thompson devoted herself to every cause which had for its object the welfare of the poor in the City of Dublin, and more especially her self-sacrificing efforts on behalf of the St. Patrick's Nurses' Home for Supplying Trained Nurses to the Sick Poor, seemed to call imperatively for some lasting memorial to her work and labour of love." The Archbishop described Miss Thompson's "life-work" with much feeling, and spoke of his thirty years' acquaintance with her. The Dublin Woman's Work Association owes much of its present prosperity to Miss Thompson's zeal and energy, which developed it until, at the present moment, it numbers about a hundred lady voluntary workers, seven Bible women, and seven trained nurses, who are established in the Protestant wards of poor law infirmaries. The fine Nurses' Home in St. Patrick's Green was another proof of Miss Thompson's energy, and eleven trained nurses, affiliated with the Queen's Institute, are tending the sick poor in their own homes. Of the various objects which were proposed as suitable memorials, a stained glass window in St. Patrick's Cathedral and a nurses' library in the Home have been selected, and it is estimated that a sum of £300 will cover both. £100 was subscribed in the course of the meeting.

### SEASIDE EXPERIENCES.

"The way we'll manage is this, ma'am. We can put a chair-bed in the dining-room, and the young gentleman must sleep there, and then the three young ladies can have the dressing-room. Their Pa and Ma will have the front bed-room, and the nurse and the children will be very comfortable in my front basement. Oh, yes; it's quite healthy. I never heard no complaints." "Well, it seems rather crowded," says the lady; "but what are your terms?" "Well, we'll say seven guineas and extras," is the bland response. And in spite of the terms, and in opposition to her own good sense, the mother agrees to exchange her roomy suburban villa for seaside lodgings of such limited dimensions and modified sanitary arrangements that only their persistent out-of-doors life saves the members of her family from serious ailments, whilst it does not prevent their all suffering from morning headaches and restless nights.

### SHORT ITEMS.

The annual sermon in aid of the funds of the St. Patrick's Nurses' Home was preached in the cathedral on the 12th inst. by the Archbishop of Dublin.—It is announced that Miss Florence Nightingale has promised to contribute a paper on "Village Sanitation in India" to the Tropical Section of the Eighth International Congress of Hygiene and Demography at Budapest. Miss Nightingale is one of the hon. presidents of the section.—Increased accommodation for the nursing staff will be secured by the new buildings already in progress at the rear of St. Mark's Hospital in City Road.

### On General Nursing.

By Rowland Humphreys, M.R.C.S., L.R.C.P.Lond.

### XXVI.—OBSTRUCTION OF THE BOWELS.

Obstruction to the passage of food or fæces through the bowel is merely one of the symptoms, and a minor one, of a number of diseases, which as a matter of chance or as a natural result close the lumen of the intestine.

Under the two clinical heads of chronic and acute obstruction may be grouped most of the forms, though there is a tendency for the chronic cases to become more or less acute, and *vice versa.*

Under the heading of acute cases may be mentioned the more common ones of strangulated hernia, intussusception, bends from adhesions, and obstruction by a band.

Under the chronic form may be mentioned impaction of fæces, stricture of the large intestine, and matting together of the coils from inflammatory or cancerous disease.

Generally speaking, the severity of the symptoms vary with the seat of the disease; the higher up the obstruction be, the more acute are the symptoms; the lower down, the more chronic.

In chronic cases there will be a history of more or less severe symptoms preceding the attack; in the acute ones the onset is sudden and unexpected—indeed, as in the case of internal bands, the cause has often been present for years without giving rise to any trouble, till by some unfortunate series of events the band restrains movement or prevents the passage of some unusually sized mass, or, as in the case of a hernia, an additional piece of intestine may have slipped down, or some flatulent distension may have overfilled the sac, closing the exit of its contents.

In all cases of obstruction of the bowels three leading symptoms are present—pain, vomiting, constipation.

The pain is usually severe in acute cases, and is continuous; in chronic cases it is more or less paroxysmal, according to the degree of obstruction and the presence of peritonitis. It varies a good deal with the case, a nervous patient suffering much more than a phlegmatic one. The amount of pain depends, too, on the quantity of opium which has been administered, restraining as it does the movements of the intestines. In chronic cases the pain varies greatly as to character and degree.

Vomiting is another marked symptom. It does not depend on peritonitis; indeed, it exists in cases in which no inflammatory mischief is present. It is probably due to nerve influences in part, and to absorption of some poisonous material from the bowel.

The bowel contains at all times a large number of bacteria, and especially the bacillus coli commune. In health this last is not noticed by its host, but no sooner does any change take place in the condition of the intestine, or of its contents (such as by constipation, or peritonitis, or obstruction) than this bacillus becomes very virulent, and the intestinal wall becomes injured in such a manner that the bacillus can penetrate it and pass on into the tissues or into the peritoneal cavity, there setting up irritation and inflammation. The bacillus there forms poisonous material, which is absorbed and gives rise to the symptoms found in such cases.

Constipation is another leading symptom. One would naturally expect that it should be present in obstructed bowel cases, but the diagnosis is not uncommonly somewhat upset by the bowel emptying itself below the obstruction. After this has taken place, if the bowel be strangulated, not even flatus passes. The vomit then soon becomes fæculent, due to the bowel tending to throw its contents upward, the natural downward movement being prevented. The earlier the fæculent vomiting comes on the more severe is the case.

The diagnosis of the form of obstruction present, on which depends the possibility of operation, is very difficult, and at the best uncertain, and an exploratory operation is often the only sure means. In acute obstruction, where there is no external hernia, the chances are always in favour of the presence of a band, and there is often a history of a previous ttack of peritonitis, the cause of the band being first formed.

In intussusception, where the bowel has become turned inside out, sucked in into itself, the tendency is for the invaginated or sucked in part to go on increasing, and at the same time it is nipped at the point where it has been sucked in, and becoming greatly swollen becomes more or less irreducible. It plugs the intestine so far as the passage of fæces goes, but from the swollen part the flow of mucus and bloodstained serum goes on downwards, which is so characteristic of these cases. The intussuscepted part has to be pushed back again, either by the injection of air or water, preferably the latter, or reduced by abdominal operation. This form of obstruction occurs, principally, in young male children, and the mortality after abdominal operation in these cases has, up to a recent date, been very great; by keeping the patient thoroughly warm during the operation and making it as short as possible, and operating early, the operation has lately become far more successful. The passage of the blood-stained mucous by the rectum, the presence of a tumour in the right iliac fossa, or even in the rectum itself, are the principal characteristics. The pain is intermittent but severe.

Chronic obstruction of the bowel is more difficult to diagnose, as a rule, than the acute form. It often exists for a long time before it is suspected, and even then the patient commonly comes late under treatment.

One form of chronic obstruction is that due to weakness of the muscles in the walls of the intestine, so that they are not able to propel their contents forward.

Another form is due to stricture of the intestine by the contraction of old ulcers, or to the growth of a cancerous tumour; or the tumour may be outside the intestine and merely compress it. The signs in these cases would be those of the cause, together with those of increasing obstruction.

This culminates, sooner or later, in total obstruction, due to the lodging of some harder mass than usual in the narrowed part. If unrelieved, peritonitis sets in, and rupture, even, of the intestine, may take place.

To relieve such a case, an artificial opening has to be made in the intestine above the diseased part, and if the condition of things is hopeless, the operation is directed, not only to relieving the obstruction but also to prevent the passage of fæcal material over the, possibly, ulcerated part. In such cases, where obstruction is of long standing, the abdomen becomes distended, and observation of the abdominal wall shows the intestines moving under it, their coils having become greatly distended, and their peristaltic action greatly increased. Vomiting is not usually marked, nor, as a rule, are the signs of peritonitis very manifest. In cases in which a minor condition of obstruction alone exists, the treatment is directed towards dilating the stricture, if it can be reached, in the direction of dieting the patient so that as little fæces (and those of a soft consistence) as possible may be formed, and to maintaining a regular and sufficient action of the bowels.

### Nursing at Crieff.

A sale of work was organised at Fintry Lodge the other day in aid of the funds of the Crieff Nursing Association. The arrangements were ably carried out, and a pleasant afternoon was spent by the visitors, £18 2s. 6d. being realised on the occasion. This sum included donations from friends of the nursing movement.

### Structure and Care of the Teeth.

III.—DETERIORATION.

It is commonly asserted that the teeth of the present generation are much more troublesome than those of the generation immediately preceding us, but probably this deterioration has not really occurred so recently. The apparent change in late years is partly because suffering begins earlier in life, partly because dental science has improved, and has made possible the successful treatment of teeth which our fathers would have got rid of with but little ceremony or remark. The deterioration was going on long before, but its extent is now realised; even the most superficial observer cannot fail to see it.

The strength and durability of the teeth must to some extent depend upon the use made of them by successive generations of ancestors. A perfectly-developed set of human teeth is rarely seen in civilized life; but in savage life, except where the practice of mutilating them prevails, it is difficult to find a defective set of teeth, except as the result of accident or violence. This can scarcely be entirely due to personal care on the part of the savage, although Mr. Stanley, the African traveller, says it is entirely owing to it. It is probably also due to the fact that the ancestors of the savage used their teeth to perform the work Nature intended them to do, and the proper development has continued through succeeding generations. His teeth are large, well formed, and have ample room in the massive jaw; whilst with us there is no room for the proper number of well-developed teeth, and the result is over-crowding and the onset of disease.

Busy people will not give the time to masticate properly, and consequently the exercise of their teeth is curtailed, their strength diminished, their development defective; they are less able to resist the action of the acid secretions of indigestion and other indirect forms of weakness and derangement.

The weakening process progresses, the teeth more and more readily yield to attacks that could not have had any effect if the normal strength had been maintained. Since the work of degeneration has gone on so long, a rapid cure cannot be hoped for, any more than a sick man can recover strength in a moment. Hence we must aim at patiently building up the strength of our children's teeth, guarding them from dangers, and, by checking the evil, prevent much suffering for them in the future.

Since the development of the temporary teeth began several months before the child is born, and is so far advanced at birth that the crowns, *i.e.*, the part which makes its way through the gum, is already covered with enamel, it is evident that the care of the teeth should begin before birth. Many women suffer from their teeth during pregnancy, and they may be affected by decay though no pain is felt.

In proportion as the mother's teeth suffer, the child's temporary teeth will be defective; and the care taken to preserve her own teeth will not only be a source of comfort to the mother herself, but will greatly improve the condition of the child's teeth.

Like other parts of the human frame the teeth must have material from which they can be built up, and unless they have a proper supply of lime salts they cannot be in a naturally healthy condition.

Under favourable conditions the teeth become more and more dense in structure. The central cavity containing the nerves and blood vessels, which nourish the tooth, become smaller because new bone is built up, and in old age the pulp cavity may even be obliterated in the crown of the tooth, only remaining in part of the root. If such increase be maintained the teeth are less likely to cause discomfort after maturity than before. If, however, the supply of lime salts to the teeth be deficient, and, at the same time, the constitutional disturbance which has interrupted the nourishment to the teeth produces too much acid in the mouth, which bathes the teeth constantly, rapid destruction of them will frequently result. This may occur in pregnancy. Plenty of lime may be taken in the food and drink, but it is only what can be digested and assimilated that is available to nourish the teeth. A woman may not be able to assimilate more than is required for her own sustenance, and it is no wonder, when greater demands are made that for a time her own bony frame suffers from a kind of starvation. Also the secretions of the mouth, especially the mucous, may become ropy or glutinuous, and acid in character. Hence it happens that the teeth are at the same time weakened by deprivation of proper nourishment, and are exposed to a strong acid which quickly finds all the weak, imperfect places in the enamel, and, if this destruction is overlooked and unchecked, the old saying that a woman loses a tooth for every child born is true. She will indeed be fortunate if she loses only one. Before very serious mischief has been done, the teeth themselves will give warning of danger. There will be a feeling of general sensitiveness to heat and cold; salt and sugar will cause discomfort, if not actual pain, and these indications should never be neglected.

All dentists appear to have a great contempt for the way in which the teeth are usually brushed, and declare that not one person in a hundred ever does it properly. An eminent authority on the subject lays great stress on the necessity for using an antiseptic mouth-wash in addition to the usual brushing, in order to thoroughly cleanse the whole mucous surface of the mouth, which must be used frequently and thoroughly. The most effective wash is one of carbolic acid, which can be easily made by mixing one teaspoonful of Calvert's No. I. carbolic acid with a pint of water and shaking well. Great care must be taken not to make it too strong or it will blister the mouth. Condy's Fluid may also be used. It is safer, not being poisonous, but not so effective, nor is the taste so pleasant to most people. This mixture should be used to wash every part of the mucous membrame of the mouth, and also as a gargle for the throat. If the teeth are sensitive to cold water the mixture can be made with warm water, but the same strength of the mixture should be used always, viz., a teaspoonful to the pint of water.

There are various preparations of carbolic acid, some delicately flavoured, to disguise the presence of the acid, which is the essential ingredient, which appears unnecessary, and although erroneously called acid there is nothing in it to injure the teeth.

If the mother has not good health her child's teeth will suffer. Hence for this reason alone she should keep up her strength, and should take a sufficient quantity of the earthy salts required for the development of bone in her daily food. In the case of delicate women a small dose of phosphites is useful, and readily assimilated. An occasional dose of phosphites, according to the strength of the child, will be found advisable, and a delicate child may continue it, with occasional intermissions, until ten or twelve years old, or until strong enough to do without it.

### Presentation.

On her departure from St. John's Hospital, Leicester Square, Miss Law was presented with a handsome brass clock, bearing the following inscription, "Presented to Sister Eleanor Josephine Law, by the Medical Staff of St. John's Hospital, Leicester Square, London, on the occasion of her leaving the hospital, as a mark of their appreciation of the kindliness and assiduity with which she has discharged her duties, and as an expression of their cordial good wishes for her future career."

### Old Time Nurses.

### III.—IMPEDIMENTS TO SAVING.

Many persons are found to wonder at the destitution to which nurses are often reduced, whose working days were lived through before any safe means of insuring a pension were within reach. Their earnings it is urged have been well above the average of female labour, and the expense of living during the conduct of a case is practically *nil*. Why is it then that they fail so constantly to make any provision for old age, and are obliged to struggle on long after a natural period of rest is reached, desperately striving to secure the underpaid situations which they are after all incompetent to fill.

Many causes combine to hinder a nurse from turning her earnings to the best account for herself, and so bring about the hopeless condition in which a very large number of successful workers find themselves in later life.

In the first place, the personal expenses of a nurse are exceptionally heavy compared with those of members of other callings. She must always be well and suitably dressed—"nice" in all her appointments; she must have decent lodgings in a fairly good street; if she has furniture she must retain her room with no reduction of rent during all the time she is absent at her cases; if she has no furniture she must pay an exorbitant rate for a furnished room. These expenses, absorbing a fixed proportion of her income, can be readily met.

But the income itself is not a fixed one, and unfortunately the nurse's expenses increase in exact proportion as work is slack. There are long seasons even in the career of the most successful private nurse when no call comes, when every effort to obtain work is fruitless, and when the little every-day necessities consume with fearful rapidity the savings, perhaps of years. It must be remembered that nurses are not as a rule very good managers; it has never been part of their business to make a little go a long way. The institutions in which they are trained afford neither a conspicuous example, nor conscientious training in economy, and in private life the purse-strings are commonly relaxed during periods of sickness, so that the nurse grows accustomed to somewhat lavish use of all she requires. The result is that in her own little housekeeping she wastes in a hundred ways without being the least aware of it, and is often completely at the mercy of her landlady.

With all these drawbacks there is money put away often a good deal. And here arises the second difficulty. The nurse begins to have a reputation for wealth among her relations. With her air of respectability, her comfortable surroundings, and apparently lucrative work, she becomes the resort of any member of her family who is ill, out of work, or "unfortunate," a word of wide meaning, extending from lunacy to convicted crime. It is no exaggeration to say that nine out of every ten nurses who are reduced to beggary in their old age, have been ruined by generosity to their family and friends. In most cases the help has been freely and lovingly tendered, and the sacrifices incurred most willingly borne; in some it is extorted by one pitiful tale after another, till patience is exhausted, but not, alas, the fatal habit of yielding. One elderly nurse, who had parted with all she possessed in times of prosperity to married brothers and sisters with large families, lamented bitterly the lack of a pension fund in her younger days. She could then with ease have made a comfortable provision for the future. But when her relations knew at any time she had money in the savings bank, they never rested till they got it from her. And this is no uncommon case. To the question, "Have you no relations who can help you?" the answer will be, with a sad shake of the head, "Indeed, the help has been all on the other side," and then will follow the usual story of earnings appropriated for the use of others, who too commonly repudiate all obligation in the later period of distress. In no way does the Pension Fund act a more useful part than in helping to block the ceaseless importunities which hinder so many hard-working women from independence.

The nurse is, in fact—all honour to the calling—very commonly the most industrious and unselfish member of her family. The instincts which led her to choose for her life-work the care of the sick, have marked her out as the one to turn to in trouble, the one on whom the feeble naturally lean. With her the invalid mother or broken-down father finds a refuge; she will strip herself cheerfully of the savings of many years to send succour to some widowed sister far away, or undertake the support of the orphaned nephew or niece. The very fact of her earnings being often far in excess of her wants is a snare to a generous and unworldly nature; she forgets that the good years are exceptional and binds upon her shoulders in seasons of prosperity burdens which sooner or later will involve her in embarrassment or dependence.

It is characteristic of the nurse that her marriage is very often a failure. Whatever be the cause, whether she marries for pity, or is married from speculation, the man turns out a *mauvais sujet*, or a hopeless invalid, or just a cypher who can never get work. The result is the same. The nurse earns for the family and spends her best years in putting the children out in the world, only to find they all marry in their turn and have large families, while the few shillings necessary for her weekly support are hard to find among them. The widow with young children, who takes to nursing for their support, is at a similar disadvantage with respect to laying by for old age. But the continual sacrifice involved, in the case of these married and widowed nurses, in paying their pension premiums, is far more than compensated by freedom in the end from the terribly painful necessity for dunning those they love best for their daily bread.

Lastly the nurse who not only saves but retains her savings falls a natural and most easy prey to bad investments. She sets up an invalid home, breaks down in health herself and loses everything. Or she joins forces in a boarding-house with a friend who becomes bankrupt and involves her in the same ruin. It is no reproach to her that where the most prudent man of business is liable to loss, in the matter of companies and business ventures, she should be miserably deceived. She is trustful to the verge of recklessness in her business dealings and has been known to confide hundreds of pounds to some acquaintance to invest in unknown undertakings, with no better title of possession than a mere letter of acknowledgment, and the assurance that the money has been secured to her in the recipient's will, in the event of his death—(fact).

The nurse is in fact notoriously ill adapted to look after herself. May she long continue to support her character for unworldliness, generosity, and disregard of self. But let her ask herself in time whether a steady systematic surrender of income, to ensure subsequent independence, would not yield a richer harvest of real good, than a reckless liberality, inevitably resulting as years go on in the miserable reversal of her position from benefactor to suppliant.

### Appointments.

Leeds Hospital for Women and Children.—Miss Ellen Mary Wreford has been appointed Matron of this institution. She was trained at the Royal Infirmary, Bristol, for three years, and then worked for three and a-half years in connection with the Leeds District Nursing Association. She subsequently obtained the post of head nurse at the hospital, which she held for sixteen months before her present appointment. We wish her every success.

### Waiting.

A nurse's life embraces a vast amount of waiting. The probationer, in the early days of her training, is often jealous of the pauses in active work in which her staff-nurse indulges; but when her own knowledge of the fitness of things develops, she learns that any serious employment, followed methodically, allows of interludes.

The nurse who has the largest experience of "waiting" is, perhaps, the one engaged in private work. She goes, at a moment's notice, into a strange household and remains there until the need for her attendance is over, when she packs up her small trunk and returns to her headquarters. Whether this is a hospital, home, or her own hired lodging, one fact is always the same, she must "wait" for the next case.

A methodical woman usually puts all her possessions into order at once. She sees to the renewal of garments which have succumbed to "wear and tear," and gives heed to the repair of her shoes, oi to the replacement of those "delightfully comfortable" old ones which she regretfully condemns as being unfit for active service. When all is in order, the experienced nurse sets herself to rest, and she often seems like the soldier whom nothing can surprise or embarrass. At the same time, in spite of knowledge and a calm demeanour, she is still often shy of strangers and new surroundings, and so her patient waiting is attended by misgivings. When the trained nurse's "waiting" is ended by a sudden demand for her services, she is quite as grateful for a kindly welcome as her patients can possibly be for the reassurance conveyed by the new comer's immediate interest in her new case!

### Progress at Northampton.

The guardians have determined to inaugurate a thoroughly good system of nursing in their workhouse infirmary at Northampton. Frankly owning that "their infirmary had needed improvement for years" they will adopt no half-measures of reform. The reports of a recent board meeting show in fact a laudable determination to carry out the recommendations of the committee selected to go into the matter and to deal with the scheme for providing skilled nursing for the sick and the aged. The appointment of a competent trained nurse from the Workhouse Infirmary Nursing Association is the first step contemplated, and doubtless the guardians at Northampton also realize the absolute need for the provision of adequate night nursing. There seems to be every hope of the arrangements in this infirmary being organised on modern as well as humane principles, and we shall be interested in further developments of the new scheme.

### Miss Kate Marsden.

A correspondence between the Rev. A. Francis, of the St. Petersburg British-American Church, Secretary to the Committee of Investigation, and Miss Marsden, which appeared in the *Times* on the 16th, 18th, and 20th inst., should receive close attention from nurses and the public. This correspondence has ended in an action by Miss Marsden for libel against Mr. Francis, which we feel must properly lead to the suspension of Miss Marsden's operations until the trial of the action.

### A Garden Fete.

In the grounds of Stagshaw House, Corbridge, a charming garden fête was recently held held on the occasion of a sale of work in aid of the funds of the Cathedral Nurse and Loan Society. Refreshments at a nominal charge were provided in a tent, and the stalls of work were placed in the conservatory. A band and games for the young folks added considerably to the success of the entertainment.

### For Reading to the Sick.

### PAIN.

**Motto.**

Pain is to save from pain; all punishment
To make for peace; and death to save from death;
And second death, to guard immortal life.

—*Edward Young.*

**Verses.**

The Man of Sorrows, and the Cross of Christ
Is more to us than all His miracles.
And that most closely we may follow Him
By suffering, have all hearts of men allowed.
Is suffering then more near and dear to God
For its own sake than joy is? God forbid!
We know not its beginning nor its end; . . . .
We suffer. Why we suffer—that is hid
With God's foreknowledge in the clouds of Heaven.
. . . . And no answer yet
Is found, nor will be found while yet we live. . . . .
But yet one thought has often stayed by me
In the night watches, which has brought at least
The patience for the hour, and made the pain
No more a burden which I groaned to leave,
But something precious which I feared to lose.

*H. E. H. King.*

Self-love no grace in sorrow sees,
Consults her own peculiar ease;
'Tis all the bliss she knows.
But nobler aims true love employs;
In self-denial is her joy,
In suffering her repose.

—*Cowper.*

**Reading.**

The Cross of Christ is the centre of all suffering; it absorbs all suffering which does not naturally flow from it; there is not one which the Cross of Jesus does not explain. My dear friends, when we recollect that Jesus Christ suffered for us; when we consider that all we suffer is a feature of resemblance with our Savour, and that from the extent of His sufferings, the more we suffer the more we resemble him. Is it not true that this modifies our afflictions? The thought that Jesus Christ suffered affiiction before we did—that it could not be spared Him—is it not at the same time enlivening and sweet? And who can be so cast down that he cannot be comforted by the thought: this is like my Savour; it is a feature of resemblance to Him. Now I know that I belong to Him, that He calls me, and that I begin to enter into the views of God and to under stand His ways, I unite my cross to His cross, and my sufferings to His sufferings.—*Adolphe Monod.*

Where is the light to lighten the darkness, the light that shall drive away the gloom of our prison? It is here. It was our Lord Jesus Christ who taught the world new lessons about pain. He it was who placed it once for all in a new light.—*Caroline M. Hallett.*

And God does not wait to be loved by His children before He loves them. He does not wait until you are better and holier. No! he begins by loving them just as they are. His love is not a reward, for no one earns His love. It is a free gift. . . . The strongest proof of God's love was Jesus Christ's coming down to earth. God gave His best; could He do more? And our Saviour taught His disciples to say "Our Father." Father means love. . . . God's love is a very real, present thing, more lasting than this world and all it contains. . . . O do believe in this love of His. Say simply as a little child, "My Father." They are words full of rest and peace. "My Father knows me yet still. He loves me. Someone stronger than I is holding me up through all my weakness and weariness." Yes, "the Father Himself loveth you."—*C. M. Hallett.*

### Character Sketches from a Lunatic Asylum.

### THE PATIENT WHO WAS HAUNTED BY SPIRITS.

SHE was really a very nice old lady, perfectly quiet and harmless, and a pleasing contrast to most of the other patients, in that she never used any bad language and never was heard to speak ill of any of the nurses. She was not far from seventy, but no one would have thought it to look at her, and she herself believed it least of all. Old, indeed! Life was yet before her. Only let her get rid of these tiresome spirits, which so constantly tormented her and everyone about her, and then she would leave the asylum and go forth into the world once more, and become a governess or a school teacher again, as she used to be in former days.

Truly, those spirits did torment her! I do not mean by spirits intoxicating liquors, which never passed her lips, but spirits of the air, minute and impalpable, yet which she herself could see, and hear, and feel. They got inside her in such crowds that her finger tips felt as if they were bursting, and they caused her such pain in her foot and leg that they often kept her awake all night. The poor old lady had really a diseased foot, which never healed, and often obliged her to lie in bed; but she put it all down to the spirits, whose antics occupied the greater part of her time.

It was not for want of conciliatory attentions that those spirits were so bent on mischief. She saved scraps of food for them from every meal, and put them outside the window; and when the other patients took them, she was quite satisfied that the spirits had eaten them. "Bring me a little piece of cake for the spirits," she would say to me when there was an entertainment given which she was unable to attend, "they are so hungry, and if I do not give them something to eat, they are so troublesome."

Moreover, she spent hours in repeating mysterious charms, in some language known only to herself, for the most part; I could scarcely ever pass through the corridor, in the daytime, without seeing the old dame hopping about, her lame foot swathed in innumerable rags, gazing heavenwards, and waving her hand as she repeated, "Up—go far away! Up—go far away!" over and over again.

Wonderful were her accounts of the performances of these spirits over whom she alone had any control. On one occasion there was a patient dying in the next room to her own. The poor woman was wasted to a skeleton, and groaned a great deal during the night. The next morning our friend informed me seriously that the reason of her groans was that the spirits had put a waggon and several oxen inside her! Sometimes the spirits let the whole asylum down into the earth and brought it up again, in the course of the night, without any of the inmates being aware of the proceeding, and, again, they would turn the building completely round, using some kind of lever for the process. The reason and the manner of these performances was carefully explained to me more than once, but I fear I am still very ill-informed on the subject. I am quite of one mind, however, with the nurse who was arrested by this old lady one day with the rather startling inquiry, "Nurse, would you mind lending your room to the devil for four nights, at a pound a night?" The nurse said she was quite willing, provided she was sure of the money; whereupon the old lady hastened to assure her that it was a good devil! I never heard that the transaction came off, after all. One thing was certain, however. As soon as the spirits had finished their mysterious occupation they would all go away, and then we should all be able to leave the asylum and go away too.

In anticipation of this happy consummation, and of the time when she should be able to take up teaching again, our old friend used occasionally to practise (!) on the piano and to try to do sums, the simplest of which seemed to baffle her. Sometimes she would appeal to me for assistance, when she used to involve me in nearly as much perplexity as herself. "You see, I don't want to forget all my arithmetic," she would say, "because by next Christmas the spirits will be all gone, and I shall go out and teach again." Many Christmases had come and gone, but the spirits were still there.

Perhaps it was the spirits that made her so fidgetty too. Certain it is that she was a perfect type of the fussy old maid. When she slept in dormitory she was the despair both of patients and nurses; of patients, because she would get up at night and prance about the room, exorcising the spirits; of nurses, because it was impossible to keep any room tidy where she was allowed to be. She must have a certain number of handkerchiefs and rags spread out on chairs to dry; she must have her boxes with all her things arranged in a certain way; she must have her papers and her needlework and a hundred-and-one indescribable articles that were necessary to her existence, or, at least, to her happiness. And as they really were necessary to her happiness, and there was no reason why she should not have them, she was finally allowed a little room to herself, which she could arrange and fidget about as much as she pleased, and where her apparatus of chairs and rags interfered with no one. She was wonderfully energetic, considering her age and her feeble health, and was always up soon after six shaking out her mats and sweeping out her own room and carrying out her clothes to air, though all the time she could never put the one foot to the ground, except just the toes.

If she wanted anything, no one in the place had any peace until she got it, for she carried out to the full the principle of obtaining by importunity what could not be obtained in any other way. If you were really too busy to stay and listen to her, you must fly—there was no other way; if once she fastened upon you with a list of her wants and grievances, there was no escape under three-quarters of an hour. Were you going out, would you buy her some hairpins, one particular kind; some elastic, one particular width; some ribbon, one particular shade—no other would do. Above all, she wanted cosmetic for her hair, to prevent it from turning grey; she could not bear to look old, and for the same reason would never wear spectacles, although so extremely shortsighted that she had to hold everything, including her food, close to her eyes. As for the cosmetic, it was a perfect nightmare to everyone, for there was only one kind in the whole world that made her hair exactly the right shade, and woe betide the nurse who brought her any other kind as a substitute! In the matter of clothes, too, it was impossible to please her. Out of twenty pairs of shoes not one would fit her, until it was discovered that she was bent on wearing fours, although she could only just get her feet into fives; then, by resorting to the simple expedient of buying a pair of fives and putting four on them, she was satisfied in this respect.

Sometimes a new and inexperienced nurse would be beguiled by the old lady into promising to make her a dress. She little knew what she undertook to do. When it came to fitting on, she had a bad quarter of an hour indeed. There was always a little piece at the back that was wrong, or a pleat or a seam that must be altered; very often it ended in the old lady making her own dress, when it was always neat, but a wonderful old-fashioned shape.

She was dreadfully afraid that her door would not be properly locked at night. "Turn hard!" she would call out, as the key went into the keyhole, for if the door were not quite fast the spirits would get in, and worry her all night.

There was another influence at work, moreover, which she took great pains to avoid. This was some species of electricity by which the insanity of the other patients was conveyed through their clothes, and through anything they used. She herself (of course) was not insane, but she quite recognised the fact that the other patients were so, and for that reason she always refused to use the same kind of soap that they did, lest she should "catch their insanity!"

In spite of her ailments, she was blessed with a very hearty appetite, which never failed her, even when she was confined to bed. She was never able to go out, on account of her foot, yet she was always cheerful and contented, except when greatly tormented by her delusions. She lived very much, I think, in the future—in that bright future when she would renew her youth, and take her place in the world once more —"Next Christmas, when the spirits will be all gone!"

A. H.

### Holidays in Japan.

(By Sister Norma.)

### II.—THE JOURNEY.

The greater part of our railway journey lay through paddy fields. At Odawara we changed our train for a steam tram to Yumoto, which was much the most tiresome part of the journey, as we had to go through miles and miles of yellow, sandy scrub, and the motors (the latest American importation) had scarcely got acclimatised, and every few minutes the smiling conductor would request the passengers to get out and assist in pulling the car back on the rails. The last part of the journey from Yumoto to Myanoshita was accomplished on foot, and this was a distance of about three miles. There were mountain kagos to be hired for those who were going to Myanoshita as invalids in search of health, but as we were only going for a holiday we preferred walking to sitting in the cramped position necessary in accommodating oneself to the native vehicle. At Yumoto the scenery became thoroughly wild and picturesque. The mountains directly above our heads (for the road became a mere mountain path) in the far distance were a complete waving mass of tall bamboo. What a refreshing sound to the weary traveller is the swishing of the wind through the bamboo groves! It always brought me back in thought to English harvest-fields when a whisper of wind is carried along over the corn, passing from ear to ear, like a great golden wave. In the wild, Scotch-looking gorge to the left of our path there appeared here and there a bright mass of scarlet or purple azaleas, and at every available point, perched on some promontory of rock stood a teahouse (native inn), with its delicate whitewood walls smiling in the sun, inviting the traveller to pass through and obtain a better view of the cataracts below.

They are most cleverly contrived, these country inns, to make it necessary for the tourist to pass right through them in order to proceed on his way. They are often built upon a rustic bridge, crossing some wonderful gorge; so we naturally rest under their roofs to enjoy the view, and the gracious little waitresses lose no time in producing a dainty bamboo tray with the "honourable tea" or "sake" in small blue bowls. For this harmless refreshment the European has to pay three half-pence, which the delightful rest is well worth, even if you do not appreciate the "honourable tea." The gorge in front extends for miles, leading from Fujiyama to the sea, and all the way its precipitous sides are crowned with the most luxurious Japanese vegetation. Where the many-coloured azaleas and camellia groves have not claimed the ground for their own, the bright scarlet japonica has spread itself over the meadows and banks, and right down by the water side the cherry trees reflect their pink blossoms in the water. As we neared our destination we had to pass through a wood carpeted with bright blue iris. I have never seen such true lover's walks and such picturesque retreats conducive to sentiment as in this lovely country in the sweet spring time, yet during the whole of my residence in Japan I never met (amongst the natives) "a lover and his lass." The Japanese prefer walking hand in hand to interlacing arms. "Courting" has no part in the life of a Japanese girl, for her marriage is generally a business matter arranged on both sides between the parents of the two contracting parties, the bride-elect having no voice whatever in the matter.

When we arrived at the door of our mountain hotel the kindly-smiling manageress on the door-step received and welcomed us, and in the background rows of comely waitresses bowed low to our "honourable selves." This hotel is divided into two portions—the batchelor's quarter, which is carried on upon distinctly native principles, and the family part. All meet in the common dining-rooms, and though the life is very primitive it is none the less delightful. We are at once told that the "honourable bath" is ready, for at Myanoshita the famous hot sulphur baths are situated. Divesting ourselves of our every-day garments, we slip into the comfortable blue and white kimono, which is to be found folded at the foot of every bed in Japan, and follow our little handmaids, in their dark blue kimonos and perpetual smiles, along cool corridors carpeted with pale golden matting, the evening sunlight streaming through the paper walls decorated with queer Japanese devices. Politeness causes each little woman to turn round every few yards and bow low, reminding me of the way a dog moves, every now and then breaking into a bark to encourage his master to step out. The wooden sliding shutter is pushed back, and in a few moments, as Mr. Clement Scott puts it, "I am neck deep in sulphur water, which is purified from all smell, and has bubbled into the bath scalding hot from the depths of the earth." The baths are made of polished wood sunk in the flo or.

### A Case for Assistance.

The appeal for £20 for this case has not met with the response it deserved. We cannot much longer find space to continue it, and yet we feel our readers have not done themselves justice by the response so far received. Will not one or two of the wealthier amongst them send us a five pound note each, and so complete the £20? Surely there are many readers able and willing to subscribe to the fund Miss Bell and Miss Farlow (of the Liverpool Homes for Aged Mariners, Egremont, Cheshire), are collecting for a nurse. We trust that the £10 still needed may be forthcoming within a fortnight. Miss Bell gratefully acknowledges 2s. 6d. from E. R. Turner; 5s. Nurse E. H.; 2s. 6d. Belfast; 5s. A. J. C. (Salisbury); 10s. Miss Asked (Gravesend); making total up to date £5 18s.

### Notes and Queries.

### Queries.

(121) *Consumption.*—Kindly give some information as to suitability of S. Africa for patient of consumptive tendency.—*Nurse Edith.*

(122) *Training.*—Information wanted as to best institution for training a young woman of thirty.—*E. B.*

(124) *Training.*—I am desirous of becoming a hospital nurse. I am eighteen, and shall be glad of information.—*Augusta.*

(125) *Ship.*—Can you tell me where to apply for a post as nurse on a ship?—*District Nurse.*

(126) *Asylum Nurse.*—Where should I apply to get into an infirmary? I am an asylum nurse.—*F.*

(127) *Midwife.*—Where can I get information about midwifery training?—*Ethel.*

(128) *Qualifications.*—Would three years in a nurses' training home be as good preparation for private nursing as the same period passed in a hospital?—*Anxious Enquirer.*

### Answers.

(121) *Consumption* (*Nurse Edith*).—We are told that phthisis, in the early stage, has been treated with marked success at Bloemfontein.

(122) *Training* (*E.B.*)—Get "How to Become a Nurse," by Honnor Morten, published by Scientific Press, 428, Strand.

(124) *Training* (*Augusta*).—You are too young.

(125) *Ship* (*District Nurse*).—Do you mean stewardess? See Hospital, June 9th, and also Answer No. 81 in June 16th.

(126) *Asylum Nurse* (*F.*)—Write to the Hon. Secretary Workhouse Infirmary Nursing Association, 6, Adam Street, Strand, London.

(127) *Midwife* (*Ethel*).—Write to the Hon. Secretary Midwives' Institute, 12, Buckingham Street, Strand, and send a stamped envelope for reply.

(128) *Qualifications* (*Anxious Enquirer*).—You should get the hospital training first, and supplement it by a year in a good private nursing home if you wish to be a thoroughly competent private nurse.

☞ For Everybody's Opinion, Book World for Women and Nurses, &c., see page ccxix. et seq.

### The Muses' Looking Glass.

### "LOURDES." *

"Lourdes," which appeared firstly as a serial in the pages of "Gil Blas," has now been issued in the customary one-volume form which characterises the French novel. This, the latest of M. Zola's works, however, is barely, if indeed at all, to be classified under the title of novel. It is rather a monumental collection of the facts and fancies which surround that small grotto in the Pyrenees where history tells us a cure for suffering humanity is ever at hand—for those, at least, who drink its healing stream in faith and prayer. The subject of the book, apart from the romance which winds through the pages, is no easy an one to handle. But the great master of realism has proved himself equal to his task, and he has brought the matured observation of the intelligent lay mind to bear upon the problem yearly set forth at Lourdes. Emile Zola describes from its commencement one of these annual pilgrimages on its journey from Paris outward. The opening scene, as here depicted, of the departure of the pilgrims in the trains which roll along the irons through the raging heat of an August day, is an unparalleled piece of writing. No detail escapes the author's microscopic observation. The hospital and the lazar house seem with one, as one reads the description of this band of suffering humanity who, racked with pain and suffering, set out for a Promised Land where pardon and cure alike await them.

It is in the "white train" that we are first introduced to the characters whom one follows through the pages of the book with an interest of an entirely novel order. There are other trains innumerable, conveving the great national pilgrimage—the 30,000 souls in all who thus flock to Lourdes; there is the "red," the "blue," the "green," the "orange," and so on, but it is the "white" train which is particularly brought before our notice. This latter is particularly set apart for the worst cases of illness—at once the most hopeless and the most hopeful—for the more incurable the case the more M. Zola points out is the hold on life stronger, the faith in cure greater.

Outside the glaring sun, inside disease and death are described—in the pell-mell confusion of the impromptu hospital ward. This is the great "white" train. Here they sit side by side—cripples, paralytics, epileptics—packed in tightly one against the other, those who can sit, mattresses for those who must lie. All about, around them, sponges, towels, clothes, packets, baskets. Moving about through all, ministering angels to the massed and suffering humanity, are the good sisters of "L'Assomption." Very pathetic it is to read of the prayers and psalms offered up by the pilgrim band as they journey on, now it is the Angelus, then the Pater, the Ave, the Credo. Through the prayers, through the noise of the wheels on the iron way, the cries of pain and torture are heard at times, from the jar of the train on the aching limbs. Such a scene is strongly and vividly brought before our mind in the early chapters of the romance. One fancies one sees the people. The mother whose eyes never leave the child in her arms, the small skeleton form which is to be brought back to life and health by one touch of the sacred water The brother consumed by disease, watched over by the sister, Elise Roquet, with her half concealed, disfigured face, and so on. But our attention is directed chiefly to the figure of a young and suffering girl, Marie de Guersauet, a child in mind and stature, a woman in years. Patient, unselfish is her trouble, she is presented to us in striking contrast to her surroundings. With her are her father, and the Abbé Pierre Formont, a young priest, who has been her friend and councillor through life. His devotion is now of a purely fraternal nature, afterwards it becomes of a stronger order, Marie clings to him, he shields her. The Abbé's tenderness and pity for the girl thus stricken down in her youth and beauty is of a passing beauty; it is the romance which runs through these pages.

So sure is Marie of her cure at Lourdes that later on she tells her friends the day and the hour when the Virgin will have mercy on her. When the Procession of the Holy Sacrament passes she says to them, "I shall be cured," and very dramatic is the description of the miracle at the very day and the hour predicted, when we read of her arising from her bed, her health restored, she standing radiant in her youth and her beauty.

And what are the actual traditions, one asks, which have given to this little town in the Pyrranean Valley a notoriety above many a capital? Mankind has flocked hither at the word of a little peasant maiden. It was in the year 1858, at the Grotto of Lourdes, called Marsabiélle, in the hollow of the rock where stands the statue now, that the Holy Virgin appeared to Bernadette Soubrons no less than 18 times.†

And the Holy Virgin said to the child, "Will you do me the favour to come for 15 days? I will make you happy, not in this world, but in the next. I want great numbers of people to come to this place." During the 15 days the Holy Virgin is affirmed to have spoken often. "You will pray for sinners, you will kiss the earth for sinners. Penance! penance! penance! Go and tell the priests that a chapel is to built here. I wish to have processions in this place. Go and drink of the fountain and wash in it. You will eat of the grass that grows close by." On March 25th the Virgin said: "I am the Immaculate Conception." Consequent upon this occurrence a commission was appointed, which at the end of three months delivered a report, a report which was awaited with impatience by the people. The commission consisted of men who were bound by oath to include none but proven and certain facts. After this publication Monseigneur Lawrence wrote a pastoral letter, wherein he affirmed that the testimony of the young girl offered all the qualities that could possibly be desired.

From that acclamation until to-day, pilgrimages to the sacred spot have never ceased. In the water bathe the sick, whatever their disease. Contagions, infections of all kinds enter the water alike.

The book is one which requires to be read, and read carefully to be understood. It abounds with so much detail, such artistic descriptions, all of which has a proper bearing on the subject under discussion. Two especial pieces of realism of unequalled force are presented to us in these pages, firstly, the material side—the mercantile value of the waters of Lourdes, are pertinently commented upon; and, secondly, in the picture given us of the dead man dipped in the Grotto—the multitude in prayer around—the realism is of no mean order. But the miracle question itself M. Zola leaves much as he commenced it. He has entered into the subject with a conscientiousness, a completeness, with which it has not been treated before, and in his romance both the priest and the scientist speak; but the particular bearing of the author's mind is evident throughout. The opportunity offered to him of pointing out the necessity of a new religion for mankind, M. Zola seizes on with a characteristic insistance. Without this latter theory, which dominates the last pages of a colossal work, "Lourdes" would have possessed perhaps a greater strength, yet a greater dignity, and had the romance been left to tell its own tale. The interest which we feel in the Abbé Fromont was sufficient in itself. But Zola has used his material in a masterly manner, and in reading the book under review, one feels rather an admirer than a critic.

* "Lourdes: Yesterday, To-day, and To-morrow." By Emile Zola. (Paris, 1894.)

† "Lourdes." By Daniel Barbé. (Translated.) (London: Burn and Oates, 1894.)

### Everybody's Opinion.

[Correspondence on all subjects is invited, but we cannot in any way be responsible for the opinions expressed by our correspondents. No communications can be entertained if the name and address of the correspondent is not given, or unless one side of the paper only be written on.]

### NURSES AT KIMBERLEY.

Miss E. G. Ritchie writes: It is only this week that I have had an opportunity of reading Dr. Bishop's first letter on the Kimberley Hospital which appeared in The Hospital of June 2nd, and I should like to make a few remarks on it, as I think it is likely to create a wrong impression in the minds of many readers. Dr. Bishop "severely criticises" the management of the hospital, and omits to mention much that might be said in its favour, from the point of view of the nurses. He should also, I think, have prefaced his criticism by explaining that he did not pretend to the authority and experience gained by a connection of several years with the hospital, as he was only appointed junior house surgeon in October or November, 1893, and succeeded Dr. Ashe as senior house surgeon a few weeks later. He must have resigned his post in April, 1894, since he was at home in time to write his letter, published June 2nd. I do not know the date of his resignation, as I was called away from the hospital on December 10th, 1893, and was not able to return. Previous to my leaving I had fourteen months' training, so that in matters of personal interest to nurses I can speak from knowledge gained by practical experience. Of the board itself I know little beyond the fact that it had periodical meetings and inspections of the hospital, and several of the nurses were on friendly terms with some members of the board and their families. That notice is taken at headquarters of "suggestions for the welfare of the nurses" was proved last year when the Warden sanctioned two alterations which some of the nurses wished for; but that happened before Dr. Bishop arrived, and he is evidently not aware of the fact, which proves the unfairness of criticising with only limited knowledge. Articles concerning the management of the Kimberley Hospital appeared in a few Colonial papers—*not* "in nearly every paper." In most cases, I believe, the criticism consisted of reprints of the original articles published in a leading Cape Town paper and comments on them. Should they lead to an official inquiry into the condition of the hospital, as Dr. Bishop thinks likely, one favourable fact will be elicited, viz., that the comfort and welfare of the nurses are not forgotten. They are well fed and well housed, each nurse having a separate bedroom and the use of a comfortably furnished common room in the Nurses' Home, a building separate from the hospital although in the same compound. A matron superintends the home and looks after the nurses, and fulfils her duties well, as any of the staff would testify. It is true we wore dresses of black woollen material, but Dr. Bishop does not seem to know that we could have them thinner for summer and thicker for winter; also that we sent them regularly to the wash. In mentioning our hours on duty, he forgets to add that the work is made lighter for the nurses by the ward boy or maid respectively attached by day and night to each ward to do the rough work. I quite agree with Dr. Bishop that the nursing of coloured patients is not objectionable, and many nurses prefer them to Europeans. It is also true that consideration for others is necessary to make Kimberley Hospital, or any other, a happy place. To attain this object, no one can do so much as the house surgeons, who, by co-operating with the matron, help to maintain a good tone and spirit of loyalty among the nurses. To Dr. Bishop's remark as to the close connection between the "private staff" and the sister in charge, I should like to add that, in forming this staff to meet the demand for trained nurses for private cases in the town and district, Sister Henrietta gave another proof of this energy and self-devotion which prompted her, in the early days of the Diamond Fields, to undertake nursing there, with the help of the then Bishop of Bloemfontein. The present Kimberley Hospital, with its seven or eight wards, and large medical and nursing staff, is the outcome of her patience and perseverance in the face of many hardships and difficulties. Without pretending that it is a faultless institution, I think I may honestly say that very good training may be had in the three years necessary to obtain a certificate. During the fourteen months I was at the hospital I was under four good nurses (head), three of whom were English-trained. Excellent lectures were given by the matron, senior house surgeon, and consultants. I shall be very glad to answer, as far as I am able, any inquiries about the Kimberley Hospital at any time. I am in no way connected with it except by the interest I take in its welfare and the grateful remembrance I have of the kind help received from matron, house surgeons, and nurses.

### KIMBERLEY HOSPITAL.

Dr. Draper Bishop, late Senior House Surgeon, Kimberley Hospital, writes: If you will allow me to comment upon Miss Blennerhassett's letter which appears in your issue of the 11th inst., I shall be greatly obliged. The importance to nurses in general of the subject under discussion must be my excuse for doing so. As Miss Blennerhassett agrees to the accuracy of the facts of my letter I need not repeat them. With regard to the wearing of black stuff dresses, there are other most important points to be considered beside the comfort of the wearer, but to justify the dress by saying that the nurses know they will have to wear it before being engaged, does not strike me as a good argument. We might justify any conditions of life on the plea that people were willing to endure them. I do not know that anyone has complained that "the rule is strict and a perhaps oppressively careful watch" kept over the nurses; certainly I have not. For a considerable period before I went to the hospital and for over two months after, there was no night superintendent at all. The buildings are in separate blocks and some distance apart, and the nurses said to be very young, so this did not strike me as excessive surveillance, but rather as culpable negligence. At other times I should say the nurses were well, but not too strictly, looked after. Without wishing to say anything personal about Sister Henrietta, whose name is certainly well known in South Africa, it is evident to those who have read the colonial papers lately that there is a widespread dislike of the management of the hospital. Many people in Kimberley will not employ the nurses on the hospital private staff. It is a significant fact that one firm of doctors alone employ as many nurses for their own cases as there are on the hospital private staff, with which they have no connection. Sister Henrietta has undoubtedly done good service in the past, but to say that the hospital has grown up under her auspices is rather over-strained. The numerous cases of accidents, of camp fever, and pneumonia in the early days of Kimberley, and also the great wealth of the place at that time, rendered a large and well-equipped hospital imperative, and its existence is therefore mainly due to these facts; and the nursing profession has of late years become popular everywhere, and in South Africa, as in England, numbers of self-denying ladies offer themselves as nurses. At the Kimberley Hospital most excellent and skilful ones are to be found. The great trouble seems to be that as soon as their training is ended they sever their connection with the hospital. Hence, although the number of applications is great, it is yet sometimes necessary to get nurses of prolonged training from England, to fill some of the best positions in the hospital. I have no wish to be considered ungallant, but I do not think that many of the probationers are "girls of 18 and 20," the average age being apparently similar to that preferred in institutions in England. Granting that Sister Henrietta is all that Miss Blennerhassett says, that does not absolve the hospital board from shirking their responsibilities by giving her absolute power over the nurses. This system is strongly condemned by the *Lancet* and *British Medical Journal*, as well as the *South African Medical Journal*.

### The Book World for Women and Nurses.

[We invite Correspondence, Criticism, Enquiries, and Notes on Books likely to interest Women and Nurses. Address, Editor, The Hospital (Nurses' Book World), 428, Strand, W.C.]

Appassionata, A Musician's Story. 1 vol. By Elsa D'Esterre-Keeling. (London: Heinemann, 1893.)

It is a literary whim of to-day to write about heroines from distant shores. Norwegians, Bohemians, Dutch, Hungarians, even American Indians have passed across the stage of book-land lately, and now we are introduced in "Appassionata" to a charming daughter of Finland. She appears first as a child-like girl of fifteen, and is described as rarely beautiful and with all the fitful fancies and waywardness of a musical genius. Into her quiet life by the old Finnish harbour comes a Russian nobleman, Count Denissow, who loses his heart to the beautiful little Finlander and proposes for her hand. Selma accepts him as a matter of course, but thinks it of much greater importance that she is now to go to school in Paris, where she will have opportunities of developing her music, than the mere fact that she is a fiancée and will become a Countess in two years' time. From the first one realises that the Count, though attracted and fascinated by her beauty, does not in the least understand Selma. He is jealous even of that music which she cares so much more for than himself. Selma postpones her marriage as long as possible, but ultimately becomes the Countess Denissow, and is taken from her beloved Paris to live in solitude with the Count on his Russian estate. Here Selma naturally becomes bored. A longing for freedom seizes her, and the excitement of appearing before the world with her wonderful gift of music. This is the one thing Count Denissow is determined that his wife shall not do, being alike jealous of the admiration she would receive, and fearful of the criticism of the public. Selma's opportunity came when the Count was called away to his dying brother. She forgets the fact that she had been practically bought by the Count to be his wife, and that therefore she should have no separate will of her own against his wishes, and listens to the advice of a certain Madame Goudourow, a musician, also, who had herself loved the Count in days gone by, and been discarded by him. This woman, unknown to Denissow, had attained an enormous and almost mesmeric influence over Selma in Paris. The result of this influence is that Selma goes to Madame Goudourow and consents to play at a public concert at Helsingfors. Count Denissow, on being apprised of his wife's movements, journeys with all speed from Russia, and appears in the concert room to be suddenly stricken with blindness, the hereditary curse of his race. This scene is most dramatically described, but it is a pity that, at this point of an otherwise beautifully told story, several people and incidents are introduced which serve rather to involve than to add to the interest of the plot. The Count refuses to have any communication with Selma or to allow her to come to him even after the birth of their son. For several years, however, the husband and wife live under the same roof, Selma having disguised herself as her son's nurse rather than be separated from him. A reconciliation is only effected after the death of the child, when the book ends with the words, "They began life anew."

A Beginner. 1 vol. By Rhoda Broughton. (London: Bentley and Son. 1894.)

This is a very dull book. The story is dull, and with just one exception the characters are dull. The "Beginner" is a young woman of position called Emma Jocelyn, who writes an anonymous novel of a realistic order, and awakes to find it improper. Her friends, in their endeavours to console her, assure her of their conviction that she did not understand what she was writing about. From this suggestion Emma extracts no comfort, and turns for sympathy to the one person whom she thinks understands her, and has a soul above seeing impropriety in a truthful picture of human life. This one person is a young journalist with whom she has engaged in a promising literary flirtation. He is described as having impossible relations who are the "sort of people who keep their lamps in their drawing rooms." Just as Emma is about to confide in him, she discovers him to be the author of a very condemnatory review of her book in which he says: "Among the milliners and 'prentices who will pasture on this masterpiece, one or two may be found silly enough to take it seriously." She had taken the story very seriously herself, and her treatment of the insulter of her work might be a warning to all critics. In the end, Emma marries another man, who bought up the whole edition of her book, and made a bonfire of it. Why she forgives him this crowning insult to her literary venture does not appear. Throughout "A Beginner" one misses the sparkle and vitality which have earned Miss Broughton's earlier novels their immense popularity. The heroines in "Nancy" and "Good-bye, Sweetheart," were flesh-and-blood young women. Emma is terribly wooden, and it is only in the delineation of Lesbia Heathcote, Emma's young married cousin, that the writer is at her best. Like all Miss Broughton's novels, it is written in the present tense, and it is hard on her that so many inferior writers should have adopted this style, and made it somewhat stale.

Time and the Player. 1 vol. By Lewis Hainault. (London: Fisher and Unwin. 1894.)

This is a very sensational addition to the Independent Novel Series. The title is, perhaps, the best part of the book, but the subject-matter is morbidly unpleasant enough to ensure the attention of a certain class of readers, who no doubt will take a keen interest in the physical and mental condition of an over-strained stock-broker. Paul Lefroy awakes one day to the fact that his brain and his business are in jeopardy. His mental condition is partly attributable to hereditary taint, and partly to some accident which would be likely to develop any latent tendency of the kind. The readers of "Time and the Player" will have to follow the unfortunate Lefroy through all his despairing efforts to keep his senses during a hazardous speculation, on the result of which depends the future of his wife and children, and of which the failure would entail utter ruin. His visit to the famous brain doctor, who tells him that it is a mere matter of time with him when he will either die or go raving mad, is a very effective, if not very probable, scene, and the doctor's personality is very well drawn. The end is most dramatic. Despite the great doctor's warning, Paul continues at his work, and awaits the result of his final coup. The speculation turns out a success, and his reputation and fortune are saved, but the same day he is found lying dead in his office after hearing the good news. There is a great deal of annoying repetition in the book which is probably the result of carelessness. Such phrases as "He twisted his throat uneasily," and repeated references to "the tawny crown" of Lady Rhoda's head occur with such frequency that one grows quite nervous for Paul's throat, and very tired of Lady Rhoda's hair. One wearies too with "slim feet" placed to advantage on drawing-room fenders.

"Time and the Player" is by no means without its good points. The characters are very naturally drawn, particularly that of Joyce, Paul's wife, who makes a charming picture in her luxurious home at St. James's, living happily unconscious of the inevitable sword hanging over her pretty head. It is to be hoped, however, that Mr. Lewis Hainault will find a less uncomfortable plot for his next story.

